# Exploring Current Concepts and Challenges in the Identification and Management of Early-Stage COPD

**DOI:** 10.3390/jcm12165293

**Published:** 2023-08-14

**Authors:** Esperanza Doña, Rocío Reinoso-Arija, Laura Carrasco-Hernandez, Adolfo Doménech, Antonio Dorado, José Luis Lopez-Campos

**Affiliations:** 1 Unidad Médico-Quirúrgico de Enfermedades Respiratorias, Hospital Regional Universitario de Málaga, 29010 Málaga, Spain; esperuli@gmail.com (E.D.); adolfo.domenech@gmail.com (A.D.); antoniodoradog@gmail.com (A.D.); 2 Unidad Médico-Quirúrgica de Enfermedades Respiratorias, Instituto de Biomedicina de Sevilla, IBiS, Hospital Universitario Virgen del Rocío, CSIC, Universidad de Sevilla, 41013 Sevilla, Spain; rocioreari@gmail.com (R.R.-A.); lauracarrascohdez@gmail.com (L.C.-H.); 3 Centro de Investigación Biomédica en Red de Enfermedades Respiratorias (CIBERES), Instituto de Salud Carlos III, 28029 Madrid, Spain

**Keywords:** COPD, early disease, definitions

## Abstract

The need to improve health outcomes, as well as disease prognosis, has led clinicians and researchers to propose new ways of identifying COPD in its earliest forms. This initiative is based on the hypothesis that an earlier intervention would have a greater prognostic impact. However, the operational definition of a patient in the initial stages of the disease is complex, and there is still no unanimously accepted definition. GOLD has recently proposed different concepts to identify COPD in its early stages, such as COPD in young people or COPD with mild functional impairment. In addition, GOLD proposes two other concepts, called pre-COPD (symptomatic non-obstructive patients) and PRISm (preserved ratio with impaired spirometry), which aim to identify the patient at risk of developing this chronic airflow obstruction. However, despite the attractiveness of these concepts, none have been taken up universally by the medical community. A universally accepted identification of how to define COPD in its early stages is necessary as a preliminary step in order to design clinical trials to find out the best way to treat these patients. This review deals with these concepts of COPD at the onset of the disease, highlighting their importance and the problems involved in identifying them as therapeutic targets in real clinical practice.

## 1. Introduction

Traditional approaches to Chronic Obstructive Pulmonary Disease (COPD) appear to have failed in terms of prevention of disease progression, deterioration of Forced Expiratory Volume in one Second (FEV_1_) or mortality. Despite the potential impact of advanced inhaled therapy [1,2] and non-pharmacological approaches [3,4], COPD continues to be a leading cause of mortality worldwide [5]. Additionally, 70% of COPD patients are diagnosed at advanced stages and 50% die approximately 3.6 years after the first hospitalization [6]. Therefore, the epidemiological data indicate that COPD continues to be a disease with a high impact in the population. Consequently, there has recently been a general call for alternative formulas that provide an opportunity to improve the prognosis of the disease [7,8]. One of these initiatives is to establish a protocol which allows us to identify the disease in its earliest stages [9]. The hypothesis behind this is that it would be possible to improve prognosis and change the natural history of the disease at these stages [10]. This proposal has a solid background for several reasons. First, it has been shown that the most important loss of lung function occurs in both younger patients under 50 years of age [11] and in patients who have not yet developed severe airflow obstruction [12,13]. There is also data showing that patients with mild COPD suffer a more rapid loss of lung function in the presence of exacerbations than patients at more advanced stages of the disease [14,15]. Second, it has been shown that we can reduce mortality risk with inhaled therapies in the earlier stages of the disease [16]. Third, non-pharmacological therapies have a greater impact during the earlier stages than they do during later, more advanced stages of the disease [17,18]. 

In this context, one of the main limitations that exists today is the identification of patients in the less advanced stages, for which the term ‘early COPD’ has been coined. However, despite the term ‘early COPD’ being widely used, its concept is poorly defined. To date, this clinical situation has been referred to interchangeably with different concepts such as ‘COPD at the initial phases of the disease’, ‘COPD in young people’, or ‘mild COPD’ without giving any suitable definition of any of these clinical situations. Recently, the Global Initiative of Obstructive Lung Disease’s (GOLD’s) 2023 document (the current version of the document summarizing the recommendations for the diagnosis and treatment of COPD internationally, available at https://goldcopd.org/, accessed on 3 July 2023) made an effort to start defining these terms [19]. These concepts are currently being validated as a proposal that would potentially impact treatment. Consequently, doubts still remain among the clinical community about their implementation in daily clinical practice. In addition, GOLD 2023 proposes other terms, such as Pre-COPD (symptomatic non-obstructive subjects) and PRISm (preserved ratio with impaired spirometry), which also need explaining thoroughly. Therefore, the aim of this narrative review is to broaden the debate on early COPD with the idea of making it easier to identify these patients for whom there are greater opportunities for therapy.

## 2. Mild COPD

### 2.1. Concept and Importance

**Figure 1:**
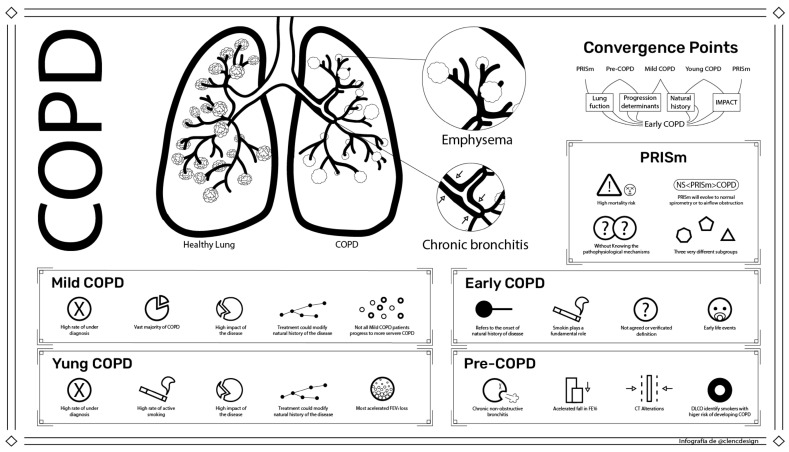
Summary of the different concepts. The figure briefly shows the main characteristics of the concepts that are reviewed by means of icons, showing the points of convergence in a visual way (see text for explanation).

The 2023 revision of the GOLD document proposes using this term exclusively to refer to the severity of how it affects the target organ, which, in the case of COPD, refers to the severity of chronic airflow obstruction measured by spirometry [19] (Figure 1). This group of patients may be relevant for three main reasons. First, it represents the vast majority of COPD patients in the community: taking into account the limitation of the high rate of under-diagnosis, according to data from the EPISCAN II study in Spain, 56% of all patients diagnosed with COPD were mild, forming the largest group [20]. Second, despite the mild airflow limitation, these patients may also suffer from a profound impact of the disease. Several studies have shown that despite this mild obstruction, patients may be symptomatic, and may have emphysema, decreased health-related quality of life (HRQoL), decreased exercise capacity and increased use of health care resources, as well as suffering recurrent exacerbations [21,22,23]. In the ECLIPSE study, 20% of the frequently exacerbating patients were patients with mild COPD [22]. These patients may also have comorbidities such as cardiovascular disease, lung cancer, or a higher risk of depression [24,25,26]. Thirdly, it may represent an opportunity for an early intervention. In the *Understanding Potential Long-Term Impacts on Function with Tiotropium* (UPLIFT) stud, the authors evaluated the response of FEV_1_ and Forced Vital Capacity (FVC) to tiotropium. They concluded that the response of these two spirometric parameters to bronchodilators decreased significantly over time and with the severity of airflow obstruction, evidencing a greater likelihood of arresting lung function loss in patients with lower degrees of airflow obstruction [27]. This is consistent with other studies showing that long-acting bronchodilators may slow the decline in lung function, as well as reduce exacerbation rates and improve HRQoL in patients with mild to moderate COPD [28]. In terms of non-pharmacological interventions, the impact is similar. Smoking cessation reduces the rate of lung function decline at all stages of the disease [29]. Interestingly, the patients with mild to moderate COPD have a higher rate of FEV1 decline compared to patients with severe or very severe COPD [14]. Similarly, there is evidence that a cohort of patients with mild COPD may suffer a more rapid loss of lung function in the presence of exacerbations than patients with more advanced disease [14,15].

### 2.2. Limitations

Although this concept is easy to measure, has clinical implications, is well standardized, and is universally accepted, certain arguments need to be clarified before opting for this form of early patient identification. COPD has traditionally been understood as a disease characterized by an accelerated decline in lung function. Consequently, it would seem logical to expect that all COPD patients would go through a first phase of spirometrically mild COPD that would be followed by a subsequent progressive decline. However, this statement may not always be true. It has been shown that not all patients with mild COPD progress to more severe airway obstruction [30]. Therefore, there are patients with mild disease that do not progress to more advanced stages even if they continue to smoke. In addition, recent studies have shown that a proportion of COPD patients experience a normal decline in lung function from a low peak lung function in early adulthood [31], and that the degree of lung function development may be impaired from an early stage of life [32]. Consequently, it would be plausible to find patients with airflow obstruction that may never be mild due to this impairment in lung function development. Consequently, the idea of COPD as a disease that starts out mild and then proceeds to progressive functional decline may not be true in either of its two premises at the patient level. Therefore, having mild COPD is not a valid way of identifying individual patients who will have accelerated lung function decline in the future.

Another issue in the debate about how to identify functionally mild patients is whether or not spirometry should be the way to identify them. However, some researchers argue that spirometry may underestimate existing physiological damage. Accordingly, other diagnostic methods are advocated, such as measuring the diffusing capacity for carbon monoxide (DLCO) [10], structural abnormalities detected in computed tomography (CT) [33], such as the thickening of the wall of segmental and subsegmental bronchi or emphysema [34], or even detecting dysfunction of the small airway through techniques such as impulse oscillometry [35].

### 2.3. Summary

Considering some of the above aspects, classifying patients by severity of airflow obstruction alone is probably an insufficient strategy [36]. Although we need more evidence on specific therapeutic interventions for this group of patients, as most large clinical trials focus on patients with severe or very severe COPD, current data show that intervening with both pharmacological and non-pharmacological treatment could modify the natural history of the disease [27,28,29], a concept which has the advantage of being easily objectifiable and clearly defined using spirometry. 

## 3. Young COPD

### 3.1. Concept and Importance

This is also a term which may appear easy to define at first, as it refers to the chronological age of the patient (Figure 1). The current GOLD proposal uses this term to refer to COPD patients aged between 20 and 50 years [19]. This group of patients is particularly important for several reasons. Firstly, because it is an age group with a high rate of underdiagnosis [20]. Second, it is a group of patients with a high impact of the disease. Notably, the available evidence shows that young patients are not asymptomatic; instead, they have a higher symptom burden, worse HRQoL, and considerably more exacerbations than older COPD subjects [10]. Additionally, there is also evidence that COPD subjects under the age of 50 years old have a more accelerated FEV_1_ loss [11,14]. Notably, some treatments, such as tiotropium, have been shown to improve HRQoL, decrease exacerbation rate and lead to a significant reduction in the decline in post-bronchodilator FEV_1_ in younger patients with COPD [11]. Third, we have data showing that among COPD patients under 50 years of age, there is a high percentage of active smokers [11]. Therefore, young COPD represents a subtype of patients which is easy to identify and has outstanding clinical consequences.

### 3.2. Limitations

Although this term is therefore attractive for clinical practice, it deserves additional comment. Firstly, its prevalence has been only sparingly studied [37]. It has traditionally been assumed that COPD begins at 35–40 years of age, with no clearly defined lower limit. However, it is possible that COPD begins earlier. However, the prevalence of classic COPD has not been studied in younger ages. In the EPISCAN II study, 4.1% of subjects between 40 and 50 years of age had COPD, of whom only 9% had been diagnosed prior to the study [20]. In addition, another consideration is the prevalence and role of alpha1 antitrypsin deficiency among this group of young patients. Although the classical phenotype of patients under the age of 50, with the presence of emphysema with progressive respiratory symptoms especially if the patient is a non-smoker or minimal smoker, may be due to alpha1antitrypsin deficiency, the reality is much more complex. Consequently, the number of patients whose alpha1 antitrypsin deficiency has been identified is currently low [38]. Another confounder in younger cohorts is the presence of asthma. We know that one of the many differences between asthma and COPD concerns the age of onset, which is earlier in asthma. Consequently, the study of chronic respiratory symptoms in a young patient should begin by evaluating a diagnosis of asthma [39]. Additionally, the combination of the two diagnoses in a single patient constitutes a challenge for the clinician, especially at younger ages, with direct clinical consequences [40,41]. Yet another consideration is that few patients in this age group have been included in large clinical trials. Despite this, several studies have shown a greater recovery rate of FEV_1_ in younger patients than in the total group of patients with the application of pharmacological treatment and preventive measures [9,11,42], while this effect is lost in older patients or those with more advanced disease [27]. Finally, the age cut-off at 50 years is arbitrary. With the general aging of the population, and the improvement in both longevity and HRQoL, the current age threshold should probably be re-evaluated.

### 3.3. Summary

In summary, although more scientific evidence is needed to support the best therapeutic alternatives for this group of patients, we do have data that suggest that taking action at this stage could modify the natural history of the disease. This definition has the advantage of being easily objectifiable and clearly defined, although this group has several disadvantages, such as having a high under-diagnosis rate, a high disease impact, and a high rate of active smoking, and not many clinical trials have been conducted on the therapeutic impact [11]. Future studies should evaluate the prevalence of COPD under the age of 50 and evaluate the impact of the therapeutic measures in the long term.

## 4. Early COPD

### 4.1. Concept and Importance

This term refers to the onset of the natural history of the disease that ends up leading to chronic airflow obstruction and its accompanying symptoms, together with an accelerated decline in lung function (Figure 1). The current hypothesis holds that COPD develops from an abrupt increase in inflammation at a specific time point, which results in airflow obstruction [43]. Early COPD seems to refer to the moment when this increased inflammation starts, if such a moment exists, which therefore corresponds to when the obstruction begins to appear, therefore making it a more biological concept.

The potential implication of identifying these patients is clear, since, if the development of the disease can be identified early on, there is clear potential for intervention. Currently, there is some evidence suggesting that this could indeed be the case. For example, several initiatives have been published on the protection of the respiratory system with antioxidant drugs [44], while others have recently started to explore study designs for potential clinical trials with these patients, who have not yet developed COPD but are at risk [42]. 

### 4.2. Limitations

Although this is extremely attractive in principle, unfortunately, it is not known when this onset of inflammation occurs, and it will probably present differently in different types of COPD patients. There are also certain challenges that make it difficult to pinpoint the exact moment. Part of this difficulty probably lies in not being able to differentiate between a true, recent onset of COPD and a persistently mild COPD that will not progress over time. To arrive at this definition would require a series of prospective cohort studies of people at risk of developing COPD of sufficient duration to demonstrate what factors ultimately influence the initiation of lung inflammation. 

In an attempt to give a more operational definition, some authors have proposed different approaches. Martinez et al. use this term for patients < 50 years old with a history of smoking > 10 pack-years and who meet one of the following criteria: FEV_1_/FVC < at the lower limit of normal, compatible alterations in CT scan or a fall in FEV_1_ ≥ 60 mL/year that is accelerated relative to FVC [45]. Other initiatives have shown that the combination of a low baseline lung function and a rapid decline in lung function (defined as an average annual fall in FEV_1_ > 40 mL) results in an increased risk of developing COPD compared to subjects with no or only one of these traits [46]. Based on these data, a functional definition of early COPD was proposed which refers to patients < 50 years old, with smoking history > 10 pack-years and FEV_1_/FVC < 0.7 (or < lower limit of normal). These were then further classified as low-activity (FEV_1_ > 50%, dyspnea measured by the modified Medical Research Council scale < 2, without frequent exacerbations and with DLCO > 80%) and early COPD with high disease activity (FEV_1_ < 50%, dyspnea mMRC > 2 and/or, > 2 exacerbations/year and with DLCO < 80%) [47]. Therefore, these definitions already include symptoms, exacerbations and tests such as DLCO. Some of these data have been corroborated in subsequent studies linking early COPD with increased symptomatic burden and exacerbations, in addition to structural abnormalities in CT and functional alterations, such as decreased DLCO [33]. Unfortunately, these definitions are research-based and have been poorly corroborated in subsequent studies. There are also authors who advocate paying more attention to early asymptomatic COPD and non-smokers, as well as focusing on early-life events such as childhood respiratory infections, exposure to air pollution and genetics [48,49,50], with the idea that the development of COPD is a long-term cumulative process, and that the development of lung function begins in the embryonic stage [51]. 

Another approach considers tobacco exposure to be the main factor to be explored. Smoking plays a fundamental role in the natural history of COPD, according to recent data. Depending on exposure to tobacco smoke, young adults with early COPD (defined as those with FEV_1_/FVC < the lower limit of normal and age < 50 years) are more likely to develop clinical COPD (defined as subjects with FEV_1_/FVC < 0.7 and FEV1 < 80%) [52]. Some studies have proposed tobacco-induced small-airway dysfunction to be the initial stage in the development of COPD [53,54], which may be detectable through techniques such as impulse oscillometry [35] or parametric response mapping techniques on chest CT that show how small-airway disease precedes emphysema [54].

Finally, from a therapeutic point of view, the effects of treatment in patients with early COPD are still unknown, mainly because we do not have an agreed upon and validated definition of this term, and there is still much to discover about the determinants that initiate the initiation. Interestingly, the evidence that early COPD is associated with poor clinical outcomes leads us to believe that early diagnosis and treatment could modify the natural history of the disease, and several studies are underway with this objective [47]. With respect to pharmacological interventions, as discussed above, there are several studies that show that treating patients with mild COPD or young COPD can modify the natural history of the disease, although none of these phases fits the definition of early COPD [47], as considered here.

### 4.3. Summary

In conclusion, the concept of biological early COPD is of great importance, because it makes direct reference to the beginning of the natural history of the disease, the moment at which it is possible to intervene to modify it. Unfortunately, although different approaches have been made to define early COPD, none of them, as mentioned above, have been agreed on or validated, and there are still many gaps in our knowledge in this respect.

## 5. Pre-COPD 

### 5.1. Concept and Importance

This term evolved from the previously used term GOLD 0, which was proposed in the first GOLD documents. It includes persons of any age with previous inhaled exposure presenting with respiratory symptoms and/or structural abnormalities and/or functional alterations in the absence of airflow obstruction on spirometry [19] (Figure 1). The concept implies that abnormalities other than spirometric obstruction, which could be clinical, functional or radiological, must be observed. 

From the perspective of clinical presentation, the definition of the term pre-COPD indicates that it refers to people with respiratory symptoms. Among these we must highlight those with chronic cough and expectoration, otherwise termed chronic non-obstructive bronchitis. These are relevant for being most commonly associated with a higher risk of COPD progression, as well as being related to worse HRQOL and episodes similar to exacerbations [55]. This subset of patients is distinguished by having a clear form of pre-COPD, as the underlying pathobiological feature (mucin production) has been identified, and they have specific structural abnormalities, such as airway wall thickening on CT [56].

In addition to symptoms, other functional impairments apart from a spirometric obstruction can also be mentioned. One of these physiological abnormalities is a decreased FEV_1_, as studies show that belonging to lower quartiles of FEV_1_, despite being within the limits of normality, is associated with an increased risk of developing COPD [57]. This term overlaps with the concept of Preserved Ratio with Impaired Spirometry (PRISm), which we will discuss later. Another functional impairment is an accelerated fall in FEV_1_, which, as previously discussed, combines a low initial lung function followed by a rapid decline, defined as an average annual fall in FEV_1_ > 40 mL, and is associated with an increased risk of developing COPD [46]. Among other functional alterations, the one that has become most important is measuring the DLCO, as it is a test capable of identifying people with a higher risk of developing COPD in smokers without airflow obstruction [56]. It has been shown that DLCO may start impairment at early stages of the disease and continues deteriorating in advanced stages of the disease, as opposed to what happens with FEV_1_ [58].

The last aspect related to pre-COPD refers to structural abnormalities. This refers to radiological alterations such as segmental and subsegmental bronchial wall thickening or emphysema, both of which are associated with an increased risk of developing airflow obstruction [34,59].

The importance of this concept of pre-COPD, in any of the forms mentioned above, is that most of these subjects are at higher risk of developing COPD, although not all of them will do so, especially if preventive measures are taken [60]. Therefore, it could represent a window of opportunity for an early intervention similar to the concept of early COPD discussed above. This has led to it being referred to recently as ‘latent COPD’ [61]. Incidentally, the prevalence of pre-COPD in people without airflow obstruction in the Spanish general population has been reported to be as high as 22.3% [62].

### 5.2. Limitations

Despite the potential for this concept, a few issues should be mentioned. One major limitation is that it confers disease status on subjects who, according to current guidelines, have not actually been diagnosed as having COPD and may never progress to the disease. Therefore, the challenge for the clinician is to determine which patients will make this transition to symptomatic clinical COPD and, therefore, which patients would be eligible for some type of therapeutic intervention for preventive purposes. Although numerous factors have already been described, such as symptoms of chronic bronchitis, decreased DLCO or alterations in the CT scan such as emphysema or bronchial wall thickening [56], much remains to be understood about the onset and progression of the disease in the patient. Secondly, with regard to therapeutic interventions that may be capable of modifying the course of the disease, apart from risk reduction measures, there have been very few therapeutic clinical trials, and there is no clear evidence of the effects that treatments might have on the natural history of the disease, although some studies have been launched in this direction [56].

### 5.3. Summary

In conclusion, this term has the advantage of presenting a clearer definition than the previous proposals, and although there are still some factors to be defined, some determinants of progression and diagnostic tests capable of detecting them have been proposed; however, we have fewer data regarding which therapeutic interventions may be capable of modifying the natural history. 

## 6. PRISm

### 6.1. Concept and Importance

This term refers to individuals with a healthy FEV_1_/FVC ratio (i.e., without airflow obstruction) but with impaired spirometry in the form of a decrease in either FEV_1_ or FVC (Figure 1). The term PRISm was initially coined for patients who are or have been smokers [14], and although many of the subjects with PRISm fall into these two categories, it has subsequently been found that the prevalence of PRISm in the general population, which ranges from 7.1 to 20.3%, is not much different from that of smokers or ex-smokers, which is around 12.3% [63,64]. Interestingly, several factors make this form of functional pre-COPD worth considering beyond its mere prevalence. Subjects with PRISm seem to have specific characteristics associated with this form of lung function impairment [65], such as frequent cases of nutritional disturbances [66,67]. There is also evidence that PRISm is associated with greater respiratory symptomatology, lower exercise tolerance and more admissions than people with normal spirometry [63,66,68]. Consequently, it is not surprising that this form of pre-COPD may be related to mortality after adjusting for comorbidities and smoking [64,66,68,69].

### 6.2. Limitations

Although this concept seems appealing, there are still many gaps in our knowledge about this category, as many of these subjects will evolve into normal spirometry and others into airflow obstruction [64,65,69], without us knowing the pathophysiological mechanisms that cause them to evolve in a certain way. What is certain is that the concept needs to be refined, since, from a spirometric point of view, this pattern can be associated with at least three different situations:

- Firstly, a preserved ratio with decreased FVC may lead to a restrictive pattern secondary to some degree of lung hyperinflation or dynamic small airway collapse in smokers [70,71].
- Secondly, this restrictive pattern could also be due to different comorbidities, such as associated pulmonary fibrosis or heart failure, and the presence of bronchiectasis or mutual or many other respiratory and non-respiratory comorbidities [72].
- Thirdly, the situation could arise where the spirometry shows a normal FEV_1_/FVC ratio and a normal FVC, but decreased FEV_1_. This latter circumstance is a spirometric pattern with uncertain consequences that should be further explored in future research.

In this regard, some authors have identified three different types of PRISm pattern [63]: PRISm-restrictive, with higher FEV_1_/FVC ratio, higher forced expiratory flow between 25 and 75% of FVC, and less emphysema and air trapping; PRISm-COPD, with average body mass index and lower FEV_1_/FVC ratio, as well as more air trapping and emphysema; and PRISm-metabolic, with higher body mass index and higher percentage of diabetics together with higher FEV_1_ impairment, lower forced expiratory flow between 25 and 75% of FVC, and thickened bronchial walls in CT [63]. Interestingly, the PRISm-COPD subgroup showed less dyspnea, better exercise capacity, measured as meters walked in the 6 min walk test, and less hypoxemia. Therefore, the authors hypothesize that this subgroup of subjects could be considered as early COPD, where the patients have not yet developed an obstructive pattern in spirometry [63]. Unfortunately, this has not been demonstrated in subsequent studies [14].

Another challenging issue regarding the concept of PRISm is whether we should maintain the fixed ratio as a signal to identify airflow obstruction or, instead, lower the limit of what is considered to be normal [63]. Such a paradigm shift would logically change the prevalence and possibly the clinical relevance of PRISm. Notably, it is important to mention that most of the studies have been carried out in Western populations. Very recently, however, new data have shown that in a Japanese population, PRISm is also related to an increase in cardiovascular and all-cause mortality and a higher risk of developing airflow obstruction [73]. 

### 6.3. Summary

In conclusion, the main advantage of this term is that its definition is based on spirometric values, and the focus is clearly on the important impact it has. However, little is known about the pathophysiological mechanisms and causes behind PRISm, and it seems to refer to at least three very different subgroups, all of which makes it difficult to establish diagnostic guidelines and design therapeutic strategies capable of modifying the natural history of the disease.

## 7. Bronchodilators in Non-Obstructive Lung Disease

The debate described above about the various ways of identifying a patient with COPD in its earliest stages makes sense only if there is a clear explanation of the therapeutic strategy to follow. Although some of the therapeutic initiatives have been discussed in this review, there is still considerable controversy over the role of bronchodilators in patients who have not yet developed an obstruction identifiable by spirometry.

There are two possible benefits of this use of the drugs evident in the available literature. Firstly, they provide symptomatic relief. It is known that COPD is a disease that has a slowly progressive onset [74]. In this clinical context, the patient usually adapts progressively to this functional limitation, in such a way that the patient may often not be aware of the symptomatic impact that their clinical situation may have. Additionally, it is possible that administering a bronchodilator treatment may lead to an improvement in the functional alterations underlying the onset of COPD leading to symptomatic relief, despite them not having an easily identifiable obstruction on spirometry.

Secondly, it is known that COPD in its earliest stages can also be associated with a situation of hyperinflation [75]. In this context, we know that long-acting bronchodilators have an effect that improves or decreases pulmonary hyperinflation [76,77], and therefore could play a role in improving this hyperinflation and consequently the symptoms, despite there not being an obstruction detectable by spirometry. Interestingly, a recent analysis explored the characteristics and bronchodilator responsiveness of early COPD patients with and without lung hyperinflation [71]. The authors described that early COPD patients with lung hyperinflation were associated with poorer lung function but better bronchodilator response. Additionally, the use of bronchodilators also has an effect on exercise capacity, prevention of exacerbations, or HRQoL [78,79] that should also be assessed in symptomatic non-obstructive cases. Consequently, some authors defend the early administration of bronchodilators [80,81].

## 8. Future Directions

**Figure 2:**
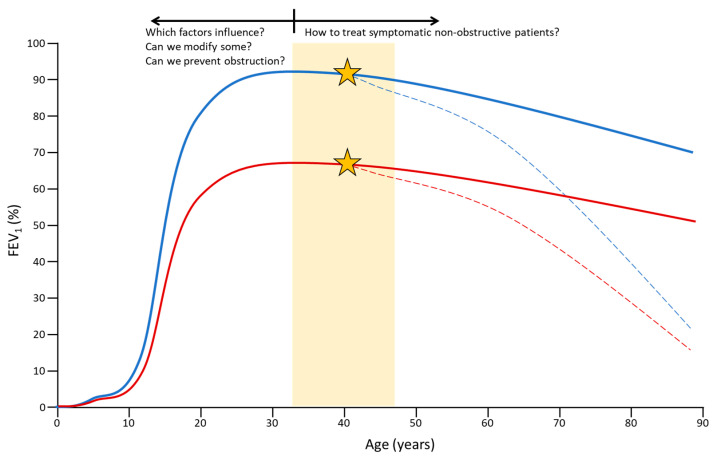
Key questions and future directions for research. The lines represent the progression of FEV1 throughout life. In blue, cases with normal lung development. In red, cases with insufficient lung development. Dashed lines represent the appearance of rapidly progressive deterioration. The star marks a point from which this progressive deterioration would begin to appear. The yellow rectangle represents what would be an age range of interest for research in adults. Graph made as a drawing on the basis of previous studies [57,84,85,86], not based on real data.

With all of the context reviewed in this document, the future lines must address two crucial aspects (Figure 2). Firstly, to advance the identification of these types of COPD in the initial stages, and secondly, to validate an agreed-upon definition that has clinical and prognostic relevance. In this sense, various authors have already proposed strategies to make advances in this direction using various instruments, from simple questionnaires or respiratory function tests to complex laboratory techniques [61,82,83]. Here, understanding well what the determinants are that influence the more or less symptomatic clinical presentation, before the obstruction appears, the factors that condition the appearance of the obstruction and the impact of modifying some of them would be research questions that should be addressed.

Secondly, future clinical trials are necessary to provide information about the efficacy and safety of long-acting bronchodilator drugs [87] or other treatment modalities in these non-yet-obstructive patients (Figure 2), who are symptomatic and may have a morphological or inflammatory alteration. 

In this sense, a working group in Spain has begun to assess an ambitious and multicenter research initiative named ANTES (anticipating the diagnosis and treatment of COPD in the 21st century) [9,10,88]. This initiative is based on the hypothesis that anticipating the diagnosis and treatment of COPD will lead to reducing the impact of the disease, improving its prevention, its treatment and its prognosis. It is probably that the results of this and other initiatives will shed new light on the resolution of the questions raised that will lead to better prevention and management of patients.

## 9. Conclusions

In summary, there is a considerable call among members of the scientific community to try to find an ideal way to identify COPD patients at the onset of their disease, given that early interventions can have a great impact on the future burden of the disease or its prognosis. The concepts reviewed here constitute an initial approximation of the different forms that COPD can have at its onset, all of which represent a window of opportunity that requires further study to find the optimal and most practical way to identify these cases in the real world. However, these initiatives are only the beginning. As soon as we decide how to accurately identify these cases, the next step will be to clarify which is the most appropriate therapeutic approach, pharmacological or non-pharmacological, that would enable a significant reduction in the burden of the disease or its prognosis. It is a complex and challenging journey, but the effort will clearly be worth it.

## Data Availability

Not applicable.
